# Relationship of attitudes toward uncertainty and preventive health behaviors with breast cancer screening participation

**DOI:** 10.1186/s12905-021-01317-1

**Published:** 2021-04-21

**Authors:** Miho Satoh, Naoko Sato

**Affiliations:** 1 Department of Fundamental Nursing, Yokohama City University, 3-9 Fukuura, Kanazawa-ku, Yokohama, Kanagawa 236-0004 Japan; 2 Department of Clinical Nursing, Fukushima Medical University, Fukushima, Fukushima Japan

**Keywords:** Breast cancer, Breast cancer screening, Mammography, Risk aversion, Health behavior

## Abstract

**Backgroundcxs:**

Early detection of breast cancer is effective for prolonging survival, but the participation rate in breast cancer screening among target Japanese women remains low. This study examined the relationships between tendencies in decision-making under conditions of uncertainty, health behaviors, demographics, and breast cancer screening participation in Japanese women.

**Methods:**

Secondary analysis was performed using data from the 2017 Keio Household Panel Survey (KHPS). The study population consisted of 2945 households. Data were obtained from the KHPS for women aged 40 years or older. Breast cancer screening participation in the past year, risk aversion, time preference, health behaviors (e.g., smoking, alcohol consumption, and medical treatment received in the past year), and demographic variables were analyzed.

**Results:**

Data from 708 women were analyzed. Among the respondents, 28.8% had attended breast cancer screening in the past year. Factors found to significantly contribute to breast cancer screening participation included higher risk aversion (odds ratio [OR], 2.34; 95% confidence interval [CI] = 1.03–5.32; *p* = 0.043), medical treatment received in the past year (OR, 1.56; 95% CI = 1.06–2.30; *p* = 0.026), higher self-rated health (OR, 1.47; 95% CI = 1.18–1.83; *p* = 0.001), living above the poverty line (OR, 2.31; 95% CI = 1.13–4.72; *p* = 0.022), and having children (OR, 1.57; 95% CI = 1.02–2.42; *p* = 0.042). Factors significantly associated with non-participation in breast cancer screening were smoking (OR, 0.20; 95% CI = 0.10–0.42; *p* < 0.000), alcohol consumption (OR, 0.56; 95% CI = 0.37–0.86; *p* = 0.007), being self-employed (OR, 0.22; 95% CI = 0.10–0.46; *p* < 0.000), and being unemployed (OR, 0.48; 95% CI = 0.26–0.90; *p* = 0.022). No significant relationship was observed between time preference and screening participation.

**Conclusions:**

The results indicate that women who recognize the actual risk of developing breast cancer or have high awareness of breast cancer prevention tend to participate in breast cancer screening. Barriers to screening participation are not working for an organization that encourages screening and low income.

## Background

Breast cancer is the most common cancer among women worldwide [1]. In Japan, it is the leading cause of cancer mortality among women aged 30–64 years [2]. Both the number of women who are diagnosed with breast cancer and the number who die of the disease is increasing; 23.4 per 100,000 population died of breast cancer in 2019 in Japan [2].

Mammography is the most effective breast cancer screening tool and can help decrease breast cancer mortality by enabling earlier diagnosis and treatment [3–5]. Since 2004, the Japan Ministry of Health, Labour and Welfare has recommended mammography screening for women aged 40 years or older every 2 years [6]. In the US in 2016, 72.5% of women aged 40 years or older had undergone mammography screening within the past 2 years [7]; the Organization for Economic Co-operation and Development reported that 70–80% of women in Western industrialized countries had undergone mammography within the past 5 years [8]. However, the participation rate among Japanese women targeted for breast cancer screening remains low. In 2016, only 44.9% of the target women had undergone mammography screening within the past 2 years [9], falling short of the targeted participation rate of 50% set in the Basic Plan to Promote Cancer Control Programs [10]. Age-standardized mortality rate in the US was 21.9 in 2010 but decreased to 19.7 in 2018 [11]. In the UK, the age-standardized mortality rate was 37.4 in 2010 but decreased to 33.3 in 2018 [12]. On the other hand, the age-standardized mortality rate in Japan was low in 2010 (9.0) compared with the US or UK, but has remained flat at 9.2 in 2019 [13]. Early detection and treatment of breast cancer can lead to favorable prognosis [14, 15]. Effective strategies are therefore urgently needed to increase the participation rate of women targeted for breast cancer screening in Japan.

Previous studies have revealed that breast cancer screening participation is associated with health-related lifestyle and behavioral factors, such as knowledge or health literacy about breast cancer [16–18], smoking [19, 20], alcohol consumption [20, 21], physical activity level [22], and self-rated health (SRH) [21, 23].

Economic factors are also barriers to breast cancer screening participation [24, 25]. According to Japan’s Census on Cancer Control [26], one of the reasons for women not attending cancer screening was “economic burden” (12.6%). Several other demographic factors are also associated with breast cancer screening participation, including higher level of education completed, lower occupational class [27, 28], regular visits to doctors [29, 30], living with a partner [31], having children [32], and older age [25, 29, 30].

Determinants of preventive health behaviors are receiving increased attention in the field of behavioral economics [33, 34]. Preventive health behaviors and the use of preventive medical care to treat fear of disease and death are likely affected by tendencies or preferences in behavior or decision-making under conditions of uncertainty [35, 36]. People often decide to use disease prevention services in consideration of intertemporal tradeoff—whether they prefer receiving an immediate small reward or a delayed larger reward—as explained by time preference theory [35, 37]. For example, the decision to participate in cancer screening is influenced by psychological value, that is, time preference as to whether to place emphasis on the present or the future.

Another tendency in behavior or decision-making under conditions of uncertainty is risk aversion. This concerns the attitude toward risk that people have when deciding to use disease prevention services to decrease the probability of disease or death [35, 36]. Even a highly satisfactory behavior may be viewed as harmful to health and thus avoided. Therefore, participation in cancer screening may be affected by an individual’s attitude toward confronting or avoiding health problems caused by cancer [35, 36]. Those who have a higher time preference tend to place value on the current situation without considering the future and therefore prefer not to engage in preventive health behaviors. Those who are likely to avoid risk tend to actively engage in preventive health behaviors [35, 36, 38]. Although decision-making tendencies under conditions of uncertainty are crucial factors that could predict preventive health behavior, there are few empirical research studies addressing this topic.

Communities in Japan are struggling with improving breast cancer screening rates. Given that women who attend breast cancer screening tend to have healthy habits, strategies have usually been proposed for people with poorer health behaviors or who are not interested in health behaviors. However, such strategies would not be fully effective considering the low rate of breast cancer screening participation in Japan. More effective tactics are needed both to encourage women to participate in breast cancer screening in order to decrease their risk of breast cancer-related health problems in the future and to improve screening participation rates.

Against this background, in this study we examined the relationship between decision-making tendencies under conditions of uncertainty and breast cancer screening participation among Japanese women. We also analyzed the relationship of health behaviors and demographics with breast cancer screening to explore more effective strategies for increasing the breast cancer participation rate in Japanese women.

## Methods

This cohort study is a secondary analysis of quantitative data from the Keio Household Panel Survey (KHPS). The KHPS survey, conducted by Keio University, provides representative data from panel surveys of Japanese households [39].

Briefly, the KHPS was approved by the Ministry of Education, Culture, Sports, Science and Technology in Japan and has been conducted annually since 2004, surveying a total of 4005 households nationwide. A stratified two-stage sampling method is used for the survey. KHPS respondents in 2004 were men and women born between 1935 and 1984. The demographic characteristics of the respondents are representative of Japanese households nationwide. The KHPS questionnaire includes items on place of residence, basic demographic data (e.g., year of birth, level of education completed, and gender), employment status, health status, health-related behaviors, and household economic status.

All participants in the KHPS were informed about the purpose of the research, potential use of their data, the survey methodology, data anonymity, strict protection of individual data, and secondary use of data. The KHPS data are collected by mail, Internet survey, and visit survey by researchers. Applications for secondary use of these data must be submitted to and approved by Keio University, with assurance provided that ethical considerations will be met. Before we started this study, Keio University approved our use of the KHPS data in accordance with our application and assurance that all ethical considerations will be met. Accordingly, Keio University then provided us with the KHPS data sets.

The empirical analysis in this study primarily used the 2017 wave of the KHPS and partially relies on the 2004 wave for basic demographic data. The sample analyzed included women aged 40 years or older, which corresponds with the recommended age for breast cancer screening in Japan. No upper limit on the recommended age for breast cancer screening has been set in Japan.

The aim of the study was to explore the relationships between decision-making tendencies under conditions of uncertainty, health behaviors, demographics, and likelihood of breast cancer screening participation in Japanese women.

### Variables

The primary outcome was breast cancer screening participation in the past year (participated or did not participate).

Explanatory variables were variables related to risk aversion, time preference, health behaviors, and demography. Risk aversion was assessed using a question about deciding whether to take an umbrella out depending on the percentage likelihood of rain in a weather forecast. Time preference is the amount of future utility that is equivalent to the current utility of consuming a good or service, and describes the preference for immediate utility (satisfaction or happiness one derives from consuming a good) over delayed utility. An individual with a high rate of time preference places more emphasis on the present and discounts the future relatively heavily [37, 40, 41]. Time preference was measured using a question about the relative value placed on smaller immediate rewards or larger later rewards [40]: “Instead of receiving JPY 10,000 one month later, at least how much would you like to receive 13 months later?” (1 “interest rate =  − 5%” to 8 “interest rate = 40%”). The KHPS asked about the following health-related lifestyle variables: alcohol consumption (yes or no), smoking status (smoker or non-smoker), weekly physical exercise (yes or no), sleep duration (hours per night), and medical treatment received in the past year (yes or no). SRH (1 “poor” to 5 “excellent”) was also evaluated. The survey also asked about the following employment characteristics: working hours per week, relative poverty (yearly household disposable income < JPY 122,000 or not), and type of employment (regular, non-regular, self-employed, or unemployed). Demographic attributes obtained included gender (male or female), year of birth, marital status (married or single), number of children (none or ≥ 1), and level of education completed (junior or senior high school, junior college or vocational school, or university or graduate school). Residential area was also recorded.

Ratios of time preference and risk aversion were obtained as percentages, with a lower time preference ratio indicating lower time preference and a higher risk aversion ratio indicating higher risk aversion. To account for relative poverty, yearly household disposable income was calculated by dividing household income by the square root of household size and then dividing participants into two groups: “living below the poverty line” (yearly household disposable income less than JPY 122,000; this is the median yearly household disposable income in Japan and is defined as the poverty line) and “living above the poverty line” (yearly household disposable income equal to or more than JPY 122,000). Age was calculated from year of birth.

### Data analysis

Frequency (percentage) and descriptive statistics (mean and standard deviation [SD]) were first confirmed for all variables. Chi-squared tests were then performed and adjusted standardized residuals (ASRs) were calculated in a preliminary analysis to compare those who participated in breast cancer screening with those who did not according to age, child status, marital status, level of education completed, type of employment, relative poverty, medical treatment received in the past year, smoking status, alcohol consumption, and weekly physical exercise. Furthermore, *t*-tests were performed as a preliminary analysis to compare differences in risk aversion, time preference, SRH, and sleep duration between the groups. Subsequently, logistic regression analysis was performed and odds ratios were calculated to identify the factors associated with breast cancer screening participation. Variables that were significant in the univariate analysis were entered into the model as exploratory variables.

All statistical analyses were conducted using SPSS Statistics 25.0 for Mac (IBM Corp., Armonk, NY, USA). Statistical significance was set at *p* < 0.05 (two-tailed).

## Results

### Demographic characteristics

In 2017, the KHPS questionnaire was distributed to 2945 households, of which 2729 responded (response rate: 92.7%). A total of 708 questionnaires had complete responses and met the inclusion criterion (answered by women aged ≥ 40 years). These responses were analyzed in the course of the present study (valid response rate: 24.0%).

Table 1 shows the descriptive statistics of the sample.

**Table 1:** Demographic data (N = 708)

	n	%
	or Mean ± SD (range)	
Breast cancer screening (during the past year)		
Yes	204	28.8
No	504	71.2
Age (years)		
40–49	170	24.0
50–59	180	25.4
60–69	183	25.8
70–79	150	21.2
80–85	25	3.5
Family type		
Living with family	622	87.9
Living alone	86	12.1
Marital status		
Married	533	75.3
Single	175	24.7
Number of children		
≥ 1	414	58.5
None	294	41.5
Level of education completed		
Junior or senior high school	449	63.4
Junior college or vocational school	135	19.1
University or graduate school	124	17.5
Type of employment		
Regular	77	10.9
Precarious	219	30.9
Self-employed	145	20.5
Unemployed	267	37.7
Relative poverty		
Poor^a^	79	11.2
Not poor^b^	629	88.8
Medical treatment received in the past year		
Yes	348	49.2
No	360	50.8
Smoking status		
Smoker	125	17.7
Non-smoker	583	82.3
Alcohol consumption		
Yes	237	33.5
No	471	66.5
Weekly exercise		
Yes	167	23.6
No	541	76.4
Region of residence		
Government-designated city^c^	199	28.1
City^d^	449	63.4
Town or village^e^	60	8.5
Sleep duration	6.52 ± 1.16 (2–11)	
Self-rated health	3.23 ± 0.91 (1–5)	
Time preference	0.17 ± 0.15 (− 0.05–0.4)	
Risk aversion	0.60 ± 0.23 (0–1)	

^a^Yearly household disposable income < 122,000 yen

^b^Yearly household disposable income ≥ 122,000 yen

^c^Population ≥ 500,000

^d^Population ≥ 50,000 to < 500,000

^e^Population < 50,000

Of the respondents, almost a third underwent breast cancer screening in the past year. Those in the age ranges of 60–69 years and 50–59 years comprised just over half of all respondents, and those aged 40–49 years comprised almost a quarter. Most lived with families; approximately three-quarters were married and over half had children. Most had not completed university or graduate school. Relatively few were regular employees, whereas more than a third were unemployed. Analysis of health behavior variables revealed that most were non-smokers, more than two-thirds did not consume alcohol, almost a quarter performed weekly exercise, and almost half had received medical treatment in the past year. Mean time preference was 0.17 ± 0.15 and mean risk aversion was 0.60 ± 023.

### Respondent characteristics according to breast cancer screening participation

Table 2 shows the characteristics of respondents in this sample according to breast cancer screening participation. Chi-squared tests and *t*-tests were performed to examine the differences in explanatory variables according to breast cancer screening participation.

**Table 2:** Respondent characteristics according to breast cancer screening participation^a^

	Breast cancer screening participation						*p*	χ^2^
	Yes (N = 204)			No (N = 504)				
	n	%	ASR^b^	n	%	ASR^b^		
Age (years)								
40–49	49	24.0	0.0	121	24.0	0.0	0.076	8.46
50–59	65	31.9	2.5	115	22.8	− 2.5		
60–69	51	25.0	− 0.3	132	26.2	0.3		
70–79	34	16.7	− 1.9	116	23.0	1.9		
80–85	5	2.5	− 1.0	20	4.0	1.0		
Family type								
Living with family	187	91.7	2.0	435	86.3	− 2.0	0.048	3.91
Living alone	17	8.3	− 2.0	69	13.7	2.0		
Marital status								
Married	161	78.9	1.4	372	73.8	− 1.4	0.153	2.04
Single	43	21.1	− 1.4	132	26.2	1.4		
Children								
≥ 1	141	69.1	3.7	273	54.2	− 3.7	0.000	13.37
None	63	30.9	− 3.7	231	45.8	3.7		
Level of education completed								
Junior or senior high school graduate	122	59.8	1.3	327	64.9	1.3	0.004	11.18
Junior college or vocational school graduate	54	26.5	3.2	81	16.1	− 3.2		
University or graduate school graduate	28	13.7	− 1.7	96	19.0	1.7		
Type of employment								
Regular	29	14.2	1.8	48	9.5	− 1.8	0.000	48.75
Precarious	93	45.6	5.4	126	25.0	− 5.4		
Self-employed	15	7.4	− 5.5	130	25.8	5.5		
Unemployed	67	32.8	− 1.7	200	39.7	1.7		
Relative poverty								
Poor^c^	11	5.4	− 3.1	68	13.5	3.1	0.002	9.61
Not poor^d^	193	94.6	3.1	436	86.5	− 3.1		
Received medical treatment in the past year								
Yes	114	55.9	2.3	234	46.4	− 2.3	0.023	5.19
No	90	44.1	− 2.3	270	53.6	2.3		
Smoking status								
Smoker	10	4.9	− 5.7	115	22.8	5.7	0.000	32.06
Non-smoker	194	95.1	5.7	389	77.2	− 5.7		
Alcohol consumption								
Yes	45	22.1	− 4.1	192	38.1	4.1	0.000	16.77
No	159	77.9	4.1	312	61.9	− 4.1		
Weekly exercise								
Yes	49	24.0	0.2	118	23.4	− 0.2	0.863	0.03
No	155	76.0	− 0.2	386	76.6	0.2		

^a^Chi-squared test and ASRs

^b^ASR: adjusted standardized residual

^c^Yearly household disposable income per month < 122,000 yen

^d^Yearly household disposable income ≥ 122,000 yen

Chi-squared tests showed significant differences in breast cancer screening participation according to family type (χ^2^ = 3.91, *p* < 0.048), child status (χ^2^ = 13.37, *p* < 0.000), level of education completed (χ^2^ = 11.18, *p* < 0.004), type of employment (χ^2^ = 48.75, *p* < 0.000), relative poverty (χ^2^ = 9.61, *p* < 0.002), smoking status (χ^2^ = 32.06, *p* < 0.000), and alcohol consumption (χ^2^ = 16.77, *p* < 0.000). Breast cancer screening participation during the past year was more likely among those who lived with family (ASR = 2.0), had children (ASR = 3.7), had completed junior college or vocational school (ASR = 3.2), had precarious employment (ASR = 5.4), had received medical treatment in the past year (ASR = 2.3), were a non-smoker (ASR = 5.7), and did not consume alcohol (ASR = 4.1). Conversely, breast cancer screening participation was less likely among those who were self-employed (ASR = 5.5) and those who were living below the poverty line (ASR = 3.1).

### Association of explanatory variables with breast cancer screening participation

The variables that were significantly associated with breast cancer screening participation in the preliminary analysis (chi-squared tests and *t*-tests) were subjected to logistic regression analysis, with age and region of residence used as control variables (Table 3).

**Table 3:** Factors associated with breast cancer screening participation on logistic regression analysis

	B	Exp (B)	95% CI		*p*
			Lower	Upper	
Age (years; reference = 40–49)					
50–59	0.36	1.43	0.86	2.39	0.172
60–69	0.16	1.17	0.67	2.03	0.582
70–79	0.13	1.14	0.58	2.23	0.701
80–85	− 0.07	0.93	0.28	3.15	0.907
Marital status (reference = single)	− 0.42	0.66	0.38	1.14	0.133
Children (reference = none)	0.69	1.99	1.20	3.30	0.008
Level of education completed (reference = Junior or senior high school)					
Junior college or vocational school	0.22	1.24	0.77	1.99	0.372
University or graduate-school	− 0.47	0.62	0.36	1.07	0.086
Type of employment (reference = Regular)					
Precarious	0.04	1.04	0.57	1.87	0.905
Self-employed	− 1.54	0.22	0.10	0.46	0.000
Unemployed	− 0.73	0.48	0.26	0.91	0.023
Relative poverty (reference = poor^a^	0.93	2.54	1.23	5.23	0.011
Received medical treatment in the past year (reference = no)	0.46	1.58	1.07	2.34	0.021
Smoking status (reference = non-smoker)	− 1.65	0.19	0.09	0.40	0.000
Alcohol consumption (reference = no)	− 0.57	0.56	0.37	0.86	0.008
Sleep duration	− 0.09	0.91	0.76	1.09	0.318
Self-rated health	0.38	1.47	1.18	1.83	0.001
Risk aversion	0.83	2.30	1.01	5.22	0.047
Region of residence (reference = Government-designated city^b^)					
City^c^	0.04	1.04	0.68	1.60	0.843
Town or village^d^	0.38	1.46	0.73	2.93	0.288
Log likelihood	− 353.879				
Probability > Chi-squared	139.74				

^a^Yearly household disposable income < 122,000 yen

^b^Population ≥ 500,000

^c^Population ≥ 50,000 to < 500,000

^d^Population < 50,000

Factors found to significantly contribute to breast cancer screening participation in the past year included being more risk averse (odds ratio [OR], 2.34; 95% confidence interval [CI] = 1.03–5.32; *p* = 0.043), receiving medical treatment in the past year (OR, 1.56; 95% CI = 1.06–2.30; *p* = 0.026), having a higher SRH (OR, 1.47; 95% CI = 1.18–1.83; *p* = 0.001), living above the poverty line (OR, 2.31; 95% CI = 1.13–4.72; *p*= 0.022), and having children (OR, 1.57; 95% CI = 1.02–2.42; *p* = 0.042). Conversely, factors found to contribute significantly to non-participation in breast cancer screening were being a smoker (OR, 0.20; 95% CI = 0.10–0.42; *p* < 0.000), alcohol consumption (OR, 0.56; 95% CI = 0.37–0.86; *p* = 0.007), being self-employed (OR, 0.22; 95% CI = 0.10–0.46; *p* < 0.000), and being unemployed (OR, 0.48; 95% CI = 0.26–0.90; *p* = 0.022). Time preference was not found to be significantly associated with breast cancer screening participation.

## Discussion

This study investigated the relationships between decision-making tendencies under conditions of uncertainty, health behaviors, demographics, and breast cancer screening attendance to explore strategies that would be more effective than those currently implemented for increasing the participation rate of Japanese women in breast cancer screening. The results showed that those with higher risk aversion tended to participate in screening. However, time preference was not observed to significantly affect participation. Women who underwent breast cancer screening were found to have healthy preventive behaviors, such as not smoking, not drinking alcohol, and having received medical treatment in the past year. Higher SRH was also associated with breast cancer screening participation. Analysis of demographic characteristics revealed that the following factors were barriers to breast cancer screening: being self-employed, being unemployed, and living in relative poverty. Furthermore, having children was positively associated with participation.

### Characteristics of the sample in this study

The rate of participation in breast cancer screening in the past year was low (28.8%) in our sample compared with that within the past 2 years in the Comprehensive Survey of Living Conditions in 2016 (44.9%) [9]. These studies have highly representative nationwide samples, but the samples are different. In addition, the time frame of the screening question was different. The present study asked about breast cancer screening within the past year, while the Comprehensive Survey of Living Conditions asked about it with the past 2 years. This difference might be the reason why the sample in the present study indicated a lower rate of participation in breast cancer screening. Analysis of descriptive statistics indicated that most participants in the present study were non-smokers and did not drink alcohol. Also, their average sleep duration was the same as that reported in the National Health and Nutrition Survey 2016 [42]. Thus, we can view these women as likely to have healthier behavior. On the other hand, the women in this study were less likely to have healthy behavior in relation to weekly physical exercise compared with data from a census on physical fitness [43] showing that 37.8–71.5% of women engaged in physical exercise on more than 1 day a week.

### Relationships between decision-making tendencies under conditions of uncertainty and breast cancer screening participation

Our results indicate that women with higher risk aversion might actively participate in breast cancer screening to decrease the risk of delayed cancer detection. There is increasing awareness that early detection of breast cancer leads to more effective treatment and thus to a better prognosis. Therefore, women with an accurate perception of breast cancer might undergo breast cancer screening to mitigate their breast cancer risk. A meta-analytic review demonstrated that perceived risk is a predictor of the likelihood of attending breast cancer screening [44]. Women who recognize the actual risk of developing breast cancer or are anxious about developing breast cancer tend to participate in cancer screening [44, 45]. Therefore, the breast cancer screening participation rate could be increased by messages that emphasize the benefits of attending screening, the necessity of screening for improving quality of life even for women with breast cancer, and the risk of breast cancer being overlooked. Also, to increase participation, it might be effective to provide appropriate health education information about the accuracy of breast cancer screening, advances in breast cancer treatment, and improvement of prognosis after treatment. However, the association between risk aversion and breast cancer screening participation has not yet been fully examined and results are not consistent across studies. Some studies have reported that women with higher risk aversion do not participate in breast cancer screening to avoid the risk of a diagnosis of breast cancer, which provokes anxiety or psychological stress [35, 41]. Moreover, Sasaki and Ohtake suggest that individuals perceive the consequences of decision-making related to breast cancer screening differently depending on whether they take a gain-framing (safety concerns) or loss-framing (risk concerns) perspective [36]. For example, on the one hand, individuals who are risk averse are unlikely to participate in breast cancer screening when considering the risky condition of “having breast cancer but its treatment might not be successful”. On the other hand, individuals who are risk-seeking are unlikely to participate in breast cancer screening because they are more tolerant of the uncertain condition of “not having breast cancer, but it might be detected.” Their survey measured risk aversion from only the perspective of loss-framing, but future studies should measure it from both the loss-framing and gain-framing perspectives to examine more effective strategies for improving the breast cancer screening participation rate.

The results of the present study also showed that time preference did not significantly affect the likelihood of breast cancer screening participation, unlike the findings of past studies [35, 36, 46]. However, a meta-analysis suggested that time preference is associated with addictive health behavior (e.g., smoking, alcohol consumption, or drug use) and not with preventive health behaviors such as attending cancer screenings or medical checkups [37]. Some studies have shown that the relationship of time preference with breast cancer screening participation is weaker than that with other factors [41, 47]. Time preference may be reflected in impulsive, addictive, or emotional behaviors rather than considered behaviors such as vaccination, medication compliance, or screening [37]. The effect of time preference might depend on mood or thoughtfulness in decision-making or decision-taking behaviors [36, 37], but remains incompletely understood. Further research is needed to clarify this effect.

### Relationship between health behaviors and breast cancer screening participation

Smoking and alcohol consumption are risk factors for various cancers, including breast cancer [48]. Past research has shown a positive association between high breast cancer knowledge and breast cancer screening participation, and suggested individuals might be more likely to undergo breast cancer screening if they are knowledgeable about breast cancer, including its risk factors, the usefulness of mammography, and causes [16, 18, 49]. In addition, individuals who are interested in preventing health problems or who actively engage in preventive health behaviors would be likely to engage more in healthy behaviors that can reduce the risk of disease or death. In contrast, individuals who engage in unhealthy behaviors such as smoking, alcohol consumption, or not attending medical checkups are less likely to engage in preventive activities [50, 51]. In particular, women who usually engage in preventive health behaviors would likely participate in breast cancer screening as a part of their preventive health behaviors [52, 53]. The results of the present study might reflect this behavioral characteristic. The survey that provided the data analyzed in this study did not ask about knowledge of breast cancer; further research is needed to clarify the associations of attending breast cancer screenings with health-related behaviors, such as smoking and alcohol consumption and breast cancer knowledge.

Several studies have demonstrated that frequent visits to medical doctors could increase the opportunities to recommend breast cancer screening and promote awareness of cancer control [21, 29, 30], whereby women who received medical treatment tended to undergo screening. The association between higher SRH and participation in breast cancer screening supports the findings of previous research [20, 54]. Individuals perceived as being in poorer physical health are less likely to attend health checkups or screenings to avoid knowing the cause of their physical health status [55]. In the present study, participants with lower SRH had high risk aversion, which might have led to their non-participation in breast cancer screening.

### Relationships between demographic factors and breast cancer screening participation

Regular employees have been shown to have the highest breast cancer screening participation rate [27, 56, 57], whereas unemployed and self-employed individuals are less likely to participate [56, 58, 59]. Employees of organizations are more likely to be exposed to recommendations for breast cancer screening or attend organized breast cancer screenings, further promoting their participatory behavior. Self-employed or unemployed women likely have limited cancer control service benefits, so they miss opportunities for screening. Self-employed women might also find it difficult to take time off to attend screenings [60, 61]. This might explain the differences in breast cancer screening participation according to employment status. Exposure to breast cancer screening opportunities or frequent recommendations for breast cancer screening is likely dependent on living environment and socioeconomic status; inequalities in either or both could lead to health disparities, and breast cancer is no exception. As such, carefully designed recommendation strategies for breast cancer screening should be jointly implemented by the government, medical facilities, and organizations. Social marketing communications could also be effective, such as combining mass media, social network services, and individual mailings.

Our analysis suggests that relative poverty should not be overlooked as a barrier to breast cancer screening. Japan has a universal health insurance system. It does not cover the cost of preventive breast cancer screening in absence of subjective symptoms of breast cancer, in which case the patient must pay out of pocket. However, the system does cover the cost when subjective symptoms are present and the doctor considers breast cancer screening necessary. As a rule, the cost of both self-funded and insured breast cancer examinations depends on the medical institution, but the cost of self-funded examinations is generally between JPY 8000 and JPY 10,000, and the cost of insured examinations is generally between JPY 3000 and JPY 5000 [62]. Income and medical insurance have been shown to affect cancer screening participation [63]. Poverty tends to delay detection of breast cancer worldwide [64], and low income tends to lead to lower breast cancer screening rates [24, 25, 65]. Free or discount vouchers for mammography have been shown to significantly improve the breast cancer screening rate in Japan [66–69]. However, there are costs involved in screening, so the provision of free or low-cost screening to targeted women should be carefully considered. Individual screening recommendations and free vouchers would likely be effective in encouraging breast cancer screening participation [70]. Strategies and educational programs should be prepared that will help women recognize breast cancer risk and the significance of attending screenings so that they can proactively participate in the screenings regardless of cost. In particular, as discussed above, support is urgently needed for women with low income and limited education. Unfortunately, Japan has no nationwide program for breast cancer screening, which is particularly disadvantageous for women in the lower socioeconomic groups and those with limited education. This study indicates that employment status and income might be triggers for non-participation in breast cancer screening. Women with these risk factors need to be identified so that they are not left out of breast cancer prevention programs. Empirical studies of the associations of breast cancer screening participation with economic status and education level are needed so that effective measures can be implemented.

Moreover, the findings of this study suggest that lower income might delay participation in breast cancer screening. Further research is needed to understand the confounders of the association between income and participation in breast cancer screening, such as educational level, accuracy of knowledge about breast cancer, accessibility of information about breast cancer screening, the difficulties in taking time off work for cancer screening and the cost burden of screening, after which effective strategies have to be built. For example, an education program about breast cancer could be implemented in primary care to ensure that no woman is without accurate knowledge of breast cancer screening. Alternatively, a public awareness campaign could be developed to ensure that all women know that screening can help prevent breast cancer.

Finally, this study found that having children is associated with participating in breast cancer screening. Women with children have childcare responsibilities, so it is likely that their health awareness is already high and that they undertake preventive health behaviors.

This study has several limitations. First, it used cross-sectional data to investigate the associations of breast cancer screening participation with decision-making tendencies under conditions of uncertainty and preventive health behaviors. As such, causality cannot be inferred. Second, the survey data were all self-reported, so the findings may have been affected by response bias [71, 72]. Further investigations are needed using objective data. Third, the study participants were from various regions of Japan, and more factors related to participants’ health status should be considered by region. Fourth, risk aversion and time preference can be measured from various perspectives, but the survey used only one questionnaire to address each of these variables. Further examination of the relationship between these tendencies and breast cancer screening participation should use several types of questionnaires to explore risk aversion and time preference. Lastly, the KHPS did not ask explicitly when respondents participated in breast cancer screening. In accordance with the recommendation to participate in breast cancer screening every 2 years, it would be necessary to ascertain whether the patients are examined every 2 years before analyzing the results of this study.

## Conclusions

This study used representative data from a panel survey of Japanese households and showed that women who recognize the actual risk of developing breast cancer and women with a high level of awareness of the need to prevent breast cancer are likely to attend breast cancer screening. Healthy preventive behaviors were also associated with participation in breast cancer screening. However, being self-employed, being unemployed, and living in relative poverty would be barriers to participation in screening.

## Data Availability

The data are available from the corresponding author upon reasonable request. The data set supporting the conclusions of this article is available from the Panel Data Research Center at Keio University with the approval of this organization (Approval No. 1322: Data ID 156-JHPS/KHPS2004-2018).

## Abbreviations

ASR: Adjusted standardized residual
CI: Confidence interval
KHPS: Keio Household Panel Survey
OR: Odds ratio
SD: Standard deviation
SRH: Self-rated health
